# Naturalistic Driving: A Framework and Advances in Using Big Data

**DOI:** 10.3390/geriatrics3020016

**Published:** 2018-03-29

**Authors:** Frank Knoefel, Bruce Wallace, Rafik Goubran, Shawn Marshall

**Affiliations:** 1 Bruyère Continuing Care, Ottawa, ON K1N 5C8, Canada; 2 Bruyère Research Institute, Ottawa, ON K1N 5C8, Canada; 3 Faculty of Medicine, University of Ottawa, Ottawa, ON K1H 8L6, Canada; 4 Department of Systems and Computer Engineering, Faculty of Engineering and Design, Carleton University, Ottawa, ON K1S 5B6, Canada; wally@sce.carleton.ca (B.W.); goubran@sce.carleton.ca (R.G.); 5 AGE-WELL NIH—SAM^3^, Ottawa, ON K1N 5C8, Canada; 6 Ottawa Health Research Institute, The Ottawa Hospital, Ottawa, ON K1Y 4E9, Canada; smarshall@ottawahospital.on.ca

**Keywords:** driving assessment, naturalistic driving study, big data analysis, framework

## Abstract

Driving is an activity that facilitates physical, cognitive, and social stimulation in older adults, ultimately leading to better physical and cognitive health. However, aging is associated with declines in vision, physical health, and cognitive health, all of which can affect driving ability. One way of assessing driving ability is with the use of sensors in the older adult’s own vehicle. This paper provides a framework for driving assessment and addresses how naturalistic driving studies can assist in such assessments. The framework includes driving characteristics (how much driving, speed, position, type of road), actions and reactions (lane changes, intersections, passing, merging, traffic lights, pedestrians, other vehicles), destinations (variety and distance, sequencing and route planning), and driving conditions (time of day and season). Data from a subset of Ottawa drivers from the Candrive study is used to illustrate the use of naturalistic driving data. Challenges in using naturalistic driving big data and the changing technology in vehicles are discussed.

## 1. Introduction

With the aging of the population [1,2], there is increasing attention being paid to older adult driving. On one hand, older adults remain healthier when they are physically, cognitively and socially active [3,4], all of which are facilitated when they stay driving [5]. On the other hand, aging is associated with decreases in vision, physical health and cognitive health, each of which affect driving ability [6,7,8], potentially increasing risk of crash and affecting their and other travelers’ health.

Current practice to assess older drivers’ cognitive driving ability includes neuro-psychological (NP) testing and observation of driving. A number of groups are suggesting a trichotomization model of triage, where NP testing is used to screen and hence reduce the number of observation-type tests. In the trichotomization model, poor NP testing is considered incompatible with safe driving, good NP results are considered compatible with safe driving, and intermediate NP results require driving observation [9,10].

Driving observation can be done in three forms. The gold standard is an on-road test with an observer in the automobile. The advantages of on-road driving include being able to observe how the driver plans their driving and reacts to traffic situations. The route can be standardized locally, allowing for more objective evaluation of driving skills. However, on-road tests are not as good at testing for unusual, higher risk events. Typically, on-road tests are cancelled in poor weather, and unsafe other driver events and pedestrian crossings cannot be “regularly” included in the test. Similarly, while some elements of on-road tests can be standardized, each city and town has unique geographic or population characteristics that prevent true standardization. Finally, on-road testing is human resource–intensive and hence costly to the individual driver or society, depending on who pays.

An alternative form of driving observation would be a driving simulator. The advantages of simulation include the ability to truly standardize a driving route and to safely include unusual high-risk events in the simulation. However, simulation cannot truly replicate all the elements of on-road driving, the driver needs to learn how to use the simulator, and the driver may be affected by driving simulator sickness [11,12].

The third method of driving observation involves naturalistic driving. Here, the older driver has the advantage of being tested in their own familiar vehicle and on roads they typically use. Another advantage is that choices around driving can be monitored. With the improvement and ongoing size reduction of technology, the older adult’s vehicle can be outfitted with cameras, global position systems (GPS), and sensors that can monitor vehicle data. Increasingly, to support driver assist and automation, manufacturers are adding sensors to vehicles, with some having standard interfaces. Since no observer is needed in real-time, this theoretically could reduce the costs associated with this type of monitoring. However, the more detailed the information one would like to collect about their driving, the more data is generated, leading to big data challenges. First the data needs to be moved from the vehicle to the place of analysis, and then there is the issue of analyzing the big data set.

## 2. Data Collection and Analysis

There are a number of driving studies that are using naturalistic driving and big data analytics [13,14,15,16,17]. The Candrive study [13,14] is one such study that included 256 drivers in Ottawa, Canada, monitored for up to five years each. On average, the Ottawa sub-group drove 20,000 km per year, amounting to a total of more than 15 M km driven. The data collected using the Persentech OttoView-CD data collection device (Persen Technologies Inc., Winnipeg, MB, Canada) attached to their car included GPS and engine computer data every second the car was driven, providing information on vehicle speed, gas pedal position, engine speed, and air temperature in addition to longitude and latitude location. In order to provide additional information about the context of the driving, this data was augmented with a Geographic Information Systems (GIS) map, weather information, and solar cycle information. To put all of this data together for analysis for the Ottawa drivers required 1 Terabyte of storage or the total amount of storage found in a typical desktop computer. The amount of processing power required to analyze each trip by each participant over the duration of the study would take all of the processing power of a typical desktop computer running continuously for about three months. However, with rapidly-evolving technology, there are continued increases in storage and computation capacity. For instance, cloud-based computing, where large-scale computation problems can be performed on shared computers from service providers, will facilitate this type of analyses.

Historically, the instrumentation of vehicles with sensors to collect driving-related data was costly, difficult, and time-consuming as the sensors and recording systems had to be retrofitted into vehicles. With the emergence of the On Board Diagnostics II interface in 1995 (for North America), many of the sensors associated with the engine became accessible through a standard interface, greatly simplifying the challenge to deploy sensors. An OBDII recorder was used in the Candrive study [13,14]. Looking forward, the number of sensors continues to increase within vehicles, and as they become accessible, they will increase the data available for study.

For illustration purposes in this paper, our group analyzed the driving data of a sub-set of 59 older Ottawa drivers that had a car crash reported to the Ontario Ministry of Transport during the period of study. The comparison of the sub-set and the full set of Ottawa drivers is shown in Table 1. As can be seen, other than gender of participant, the subset is a representative sample of the larger group.

The storage, distribution, and sharing of the collected data needs to consider the privacy of the driver [18]. Although it is relatively easy to de-identify the data by ensuring it does not include the driver name, address or other text based identity, with the inclusion of GPS information, it is relatively easy to determine the driver’s residence (where the car is overnight) and many other personal attributes that could lead quickly to the driver’s identity. Our group has proposed methods to de-identify this type of data [18].

## 3. Naturalistic Driving Framework

Given the variety of data available in naturalistic driving studies, it is important first to organize groups of information to be analyzed. Table 2 shows four categories that appear to work well: (1) driving characteristics, (2) actions/reactions, (3) destinations, and (4) conditions. These categories interact in the complex task of actual driving, but it is possible to separate them from the data to simplify comparisons.

**Figure 1:**
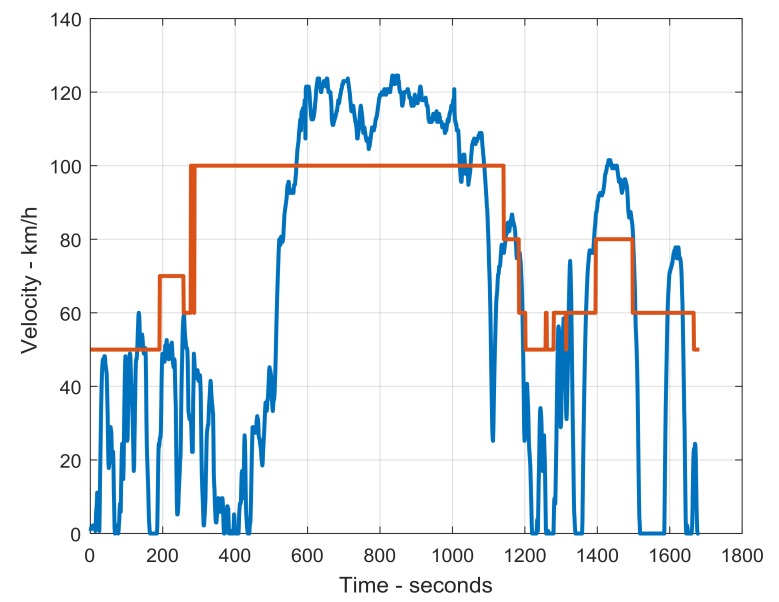
Driving speed (blue) as compared to posted speed (orange) of a trial trip by one of the authors (B.W.).

Driving characteristics that can be measured for an individual begin with quantitative measures on how much the individual drives. Two dimensions make up this measure: the number of trips taken and the length of the trip. When linked to the date and time data, distance travelled can be calculated at a trip level and on a periodic level (e.g., time of day, seasonal, yearly). The second set of characteristics relates to how the person actually drives, and this includes speed and its derivative acceleration. Speed and acceleration are relevant in both straight line travels as well as in turning situations and can be compared to the posted speed limit on a given road. Position, the third measure, includes the position in the driving lane as well as position with respect to other vehicles (e.g., following distance). Finally, the type of road used can be identified combining the GPS and GIS data. Figure 1 shows an example of a graph of actual speed travelled as compared to posted speed.

A further exploration of driving behaviors can be found through exploration of specific driving actions that are important to safe driving, including lane changes, approaches to intersections, passing vehicles, turning left into oncoming traffic and merging into traffic from a ramp. A safe driver also needs to be able to react to changes in the driving environment, such as traffic light changes, other vehicles entering their path, pedestrians crossing the road, and the rare crash or close-call event.

**Figure 2:**
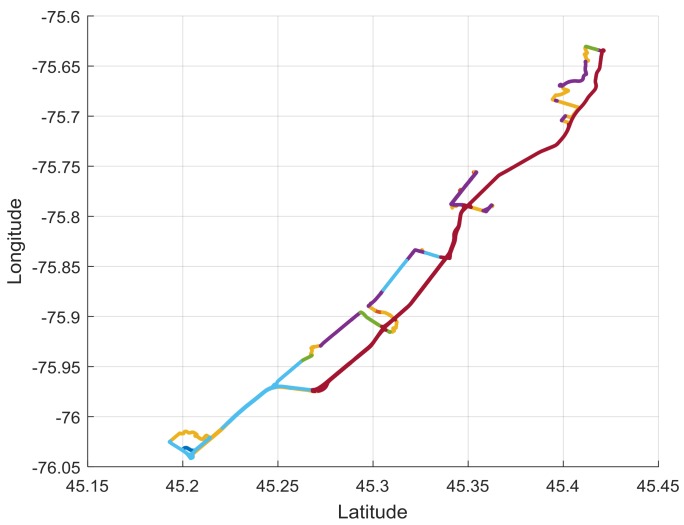
Single day of driving by one of the authors (B.W.) showing the choice of roads through the posted speed limits. (Dark Blue: not road, orange: 40 km/h, yellow: 50 km/h, purple: 60 km/h, green: 70 km/h, light blue: 80 km/h, red: 100 km/h).

The analysis of trip level or trip sequence information leads to another category of driving measurement and decision making, as the destinations chosen by the driver can be analyzed. How many different destinations does the driver have and how far are the various destinations from each other and from home. Sequencing of destinations on a given day or within a few days can be valuable to study as well, allowing, for instance, calculations of driving efficiency. Figure 2 shows a sample day of driving with the type of road chosen superimposed.

Finally, all of these driving characteristics can be further categorized through comparison to environmental/external factors, including time of day, which allows identification of daylight and traffic density (i.e., rush hour), and weather, which includes seasons and actual road conditions (e.g., wetness, ice, snow). Time of day and weather conditions can have a dual effect on drivers: first, do they make decisions to drive or not related to these conditions, and second, do they adapt their driving characteristics accordingly?

## 4. Naturalistic Driving Results

### 4.1. Distance Travelled

The foundation measure of driving is related to the amount of driving. Prior to sensor-based measurement methods, this measure was performed using periodic odometer readings, leading to a measure of driving since the last recording. This was enhanced with the use of log-based systems to try to get an indication of frequency of driving. However, by definition, these measures are imprecise because of recall challenges, forgetting to log data, or forgetting to differentiate who was driving [19]. Through the use of sensor-based measurement methods, very detailed information on distance and duration of each trip can now be acquired [19,20]. Furthermore, this level of detail allows for precise measurements of frequency of trips (trips/day) and lengths of the individual trips (km/trip), which can be used to characterize an individual’s driving preferences and allow for their monitoring over time. Decreased driving distance in older adults could correlate to changes in physical or cognitive health, either directly or by self-regulation. Table 3 shows that the sample of convenience of Ottawa drivers with crashes showed a decline in total distance driven per year over the five years of the study.

### 4.2. Speed/Acceleration

Driver speed can be calculated using both the sensor attached to the engine computer and the speed calculated using the GPS. In addition, using the speed and time data allows the calculation of acceleration, which can be compared to the sensor information on the gas pedal position. Aggressive acceleration or deceleration can include hard/emergency stops [21,22,23]. The analysis of accelerations and decelerations has been shown to provide indications of behaviors that are distinctive between drivers [24,25] and changes in acceleration patterns again could reflect health changes. In addition, the speed data can be combined with Global Information Systems (GIS) data to provide information on road type, posted speed limit and other driving hazard/warnings such as school zones. The result is a rich data set that can now be analyzed to identify specific driving behaviors and measures of specific driving maneuvers. For instance, it is possible to determine if the driver typically travels above or below the posted speed limit and if they slowdown in school zones. Again, over time, a change in driving characteristics, such as increasingly driving below the speed limit, may be related to aging or changes in physical or cognitive health.

### 4.3. Destinations

Trips can also be analyzed to locate the destinations. Destinations include both the start and ending location and locations within a trip where the vehicle stopped for an extended period (but was left idling). Examples of destination-based analyses that are possible include measures of the frequency of occurrence of the various destinations, the distance each of the destinations are from home and the diversity of destinations [21,26]. Drivers with physical or cognitive health changes could take simpler trips by choosing destinations that are closer to home. Similarly, they may reduce destination diversity, suggesting a decreased ability to learn new destinations and maintain less frequently travelled destinations. Another measure of destination-based driving behavior that is now possible using data analysis is trip sequences within a day. Over time, drivers may reduce complexity of trips by shifting from multi-stop trips to trips where they leave home multiple times to single destinations. Drivers may also shift the actual destinations that they chose such as shopping at a single general store that is closer to home instead of more complex trips going to a wider group of specialty stores. A shift to more frequent and short trips could be an indication of a health change, such as memory decline, where they have to return to the store more frequently for forgotten items. The navigational choices made for the trips can also provide insights. Although it would not be expected that drivers would always take the fastest route between two locations, a change in the chosen route could be an indication of health concerns, such as avoidance of a highway or other higher-risk roads. The Ottawa sample in this study decreased the number of trips on freeways and the total number of trips over 50 km during the time of the study (Table 3).

### 4.4. Conditions

Date, time of day, and GPS location information can also be used to determine details about the specific conditions at the time of the trip. For example, the driver may avoid higher traffic periods such as morning or afternoon rush hours. The location information, along with time of day, allows for the solar cycle to be determined. This allows the categorization of trips between daylight driving and night, dawn, or dusk when reduced visibility can increase risk. Databases from local weather authorities, which provide daily or even hourly historical records of weather events, can be used to determine the drivers exposure to trips during poor weather (snow, rain, etc.) or if they are choosing to avoid driving during inclement weather events. The sample of older drivers in this paper typically showed less driving in winter months (December to March), since 27 to 28% of their driving occurred in 33% of the calendar year. In addition, there was a significant decline in winter driving over the five year study (Table 3).

### 4.5. Driver Identification

One of the challenges of using driving data from a vehicle is driver identification. In order to determine typical driving characteristics for a driver, or their driving signature, data from other drivers using that vehicle will interfere. Many older adults live with a spouse and may share a vehicle. Even those living alone may have others drive in certain circumstances. For instance, if traveling in their vehicle with their adult children, they may allow them to be the driver. Thus, to be able to have clean data, data from other drivers needs to be removed from the data set. Interestingly, all of the driving variables that can be used to describe driving behavior may also have an important role in removing the unwanted data. Our group has shown that the analysis of accelerations and decelerations can provide indications of behaviors that are distinctive between drivers [24,25], where 91 driver pairs were analyzed and the driver of over 90% of trips was correctly identified. With the addition of other variables, the performance was improved to over 95% for some driver pairs [27]. Ongoing work in this area will likely continue to improve the probability of correct driver identification [28].

## 5. Discussion

One way to measure driving ability is a naturalistic driving study. This type of study uses the person’s vehicle and usual driving patterns and routes for data collection and analysis. Naturalistic driving studies will soon allow clinicians to evaluate specific driving characteristics as well as choices around destinations and driving conditions. This unobtrusive and potentially cheaper type of observation will likely be more acceptable to older adults than formal driving tests and driving simulators. For this to occur, the clinician will have to be provided with a summary report of the knowledge from the driving study and not the raw driving data. Big data analytic techniques now have the capacity to extract clinically useful information out of big data sets. These data could be compared to norms over shorter periods of time to compare a given driver to their peers. In addition, a baseline of their driving ability and typical behaviors can be formed. Ongoing and future work needs to focus on the identification of driver behaviors that are indications of possible health changes [29]. Monitoring driving over longer periods would allow the identification of changes in driving ability for a given driver. Identification of these changes may help older drivers make changes to driving choices, e.g., rush hour driving and night driving, could lead to targeted training interventions and could eventually lead to decisions on driving cessation [30].

Interestingly, since driving is one of the more complex instrumental activities of daily living, some groups are suggesting that driving could be used as a measure to identify and monitor functional impairment associated with aging and physical and cognitive decline [31,32,33]. In the near future, analysis of driving changes may help clinicians to categorize cognitive decline as mild cognitive impairment or early dementia. It may be that changes in driving could be detected earlier than changes on some cognitive measures currently being used.

A significant number of challenges remain before naturalistic driving can be used clinically. First of all, the optimal choice of sensors to use and the frequency of measurement have not been determined. For instance, the use of cameras greatly enhances the quality of information that can be collected. However, not all drivers may be willing to give up the privacy associated with camera use and video takes up significant volumes of data. This data volume question is also relevant for the collection of the other driving data. On one hand, the more frequently data are collected, for instance every second (as in this study), the more precise the calculation of variations in speed and acceleration, and precise location. If data were collected every minute, there would be a 60-fold decrease in the volume of data collected, but calculations of speed and acceleration would be much less precise. 

These data need to be stored and eventually transported to the place the data analysis happens. Data can be moved from the car manually 60 times more frequently or sent wirelessly at 60 times the unit cost. However, the continued expansion in the capacity of both storage devices and communication networks can quickly reduce the cost and other challenges associated with using higher-frequency sampling. Additionally, the increasing use of on-board computers will allow some pre-processing in-vehicle, further enabling higher sampling frequencies without needing to transport all the raw data.

Furthermore, a number of driving parameters are difficult to measure with recently used technologies. For instance, GPS and GIS data are not precise enough to determine lane position or position with respect to other vehicles. However, the proximity and lane position sensors currently being included for driver assist functions and adaptive cruise control could provide an alternative source of this information. While acute deceleration, for instance slamming on the brakes, can be determined from naturalistic driving data, the circumstances around the event would require additional information, such as video and likely information from other local vehicles. More work needs to be done to determine which sensors would be required and at what frequency they should be sampled.

The key limitations to long-term studies of a large number of drivers will include the challenge of collecting the data from the vehicles, processing the captured data, and the diversity in the sensor information that is available. The latter will continue to grow as newer vehicles continue to increase the number and types of sensors embedded in vehicles. In the short term, studies may have the challenge that there is too much variability in sensors being used affecting the quality of the data collected. This issue is also impacted by the renewal rate of the vehicles across the target population. The data collection solution can now take advantage of wireless networking including doing data uploads overnight using a residence Wi-Fi or even hot spots supported as needed by cellular-based data communications. Again, the emergence of cloud-based computing will allow the processing of the information to be run within computation that is shared, providing the scale needed while avoiding the cost of dedicated systems.

The increasing level of sensors within cars for automation will be both helpful and create additional challenges for future naturalistic driving studies. The addition of sensors that identify the location of the vehicle in the lane and measure the distance to the car in front are available in some vehicles today. The data from these sensors could easily be integrated into data sets to provide additional useful driving information. Again, this will complicate the data volume issue. Furthermore, many of these sensors are being used to improve safety. For instance, the sensors determining position in the lane will indicate drifting by shaking the steering wheel or even self-correcting the lane position. While these features will provide additional safety, they will decrease the ability of the driving data to help identify changes in true functioning of the older adults. In fact, these studies will likely not be correctly called “naturalistic” in the future if the vehicle has working “automation”.

## 6. Conclusions

Naturalistic driving studies are now increasingly possible given the rapid evolution of sensors and computers in vehicles. A number of driving parameters can be easily extracted from these data through the use of 1 Hz or even higher frequencies. These include driving characteristics such as number and distance of trips, speed and acceleration, position on the road, and choice of road type. Actions and reactions can also be measured from these data. When these data are combined with other readily available data, such as GIS and weather, choice of destination and driving conditions can be analyzed. Collecting these data will allow comparison of a driver with their peers and monitoring of driving change over time. Driving, as an instrumental activity of daily living, could be a measure of aging or physical and/or cognitive decline. The data extracted can also benefit the older driver by helping them choose to improve certain aspects of driving, to change their driving choices, or ultimately to decide to cease driving. Work in this area needs to continue in order to adapt to the increasing number and type of sensors and the parallel increasing amounts of data generated. Issues around data analysis and privacy will also continue to present challenges.

## Figures and Tables

**Table 1:** Demographics of convenience sample of older drivers.

Characteristic	Drivers with Crash (*n* = 59)	Total Ottawa Set (*n* = 256)
% Female	44.1	35.9
Average Age	76.3 (4.13)	76.3 (4.49) ^1^
Av. Age licensed	18.7 (4.12)	19.4 (5.83)
Av. # Yrs driven	57.7 (5.99)	56.8 (6.92)

^1^ All numbers expressed as Mean (Standard Deviation).

**Table 2:** Driving parameters.

Characteristics	How much do they drive?	Number of trips
		Distance per trip
		Distance per time of day
		Distance per season
		Distance per year
	How they drive: Speed/acceleration?	Straight/turning
		Vs. posted speed
		Vs. initial/final velocity
	How they drive: Position on road?	Position in the lane
		Distance to other cars
	Where do they drive?	Residential roads
		Local roads (50–70 km/h)
		Undivided highway (80–90 km/h)
		Divided Freeway (100 km/h+)
Actions/Reactions	Actions	Lane changing
		Approaching intersections
		Passing
		Turning left into traffic
		Merging
	Reactions	Traffic light changes
		Other vehicles
		Pedestrians
		Crash risk
Destinations	Variety and distance	
	Sequencing	
	Route planning at trip sequence level	
	Navigation within a trip	
Conditions	Time of day (e.g., rush hour, daylight)	
	Season/Weather	

**Table 3:** Driving characteristics of older drivers over five years.

Characteristic	Year 1 (*n* = 59)	Year 3 (*n* = 47)	Year 5 (*n* = 35)	*p* (Years 1–5)
# Trips	1510 (150)	1390 (518)	1210 (463)	**
Dist/trip (km)	9.44 (3.46)	8.81 (2.95)	8.83 (2.91)	
Dist/year (km)	14,000 (6660)	11,800 (5240)	10,500 (4630) ^1^	**
# Trips Freeway	1810 (1380)	1690 (1470)	1470 (1180)	
# Trips 50+ km	38.4 (48.0)	24.5 (23.3)	21.9 (12.2)	*
# Trips Night	110 (89.2)	90.0 (85.9)	75.6 (59.2)	*
Night Dist (km)	1100 (846)	925 (791)	791 (577)	*
Winter Dist (km)	4060 (2950)	3360 (2380)	2840 (1780)	*
Winter %	28.0 (10.8)	27.2 (7.5)	27.0 (12.2)	

^1^ All numbers expressed as Mean (Standard Deviation). * *p* < 0.05, ** *p* < 0.01.
